# Mimic Nature Using Chemotaxis of Ionic Liquid Microdroplets for Drug Delivery Purposes

**DOI:** 10.3390/molecules27030786

**Published:** 2022-01-25

**Authors:** Kobra Khodarahmian, Alireza Ghiasvand

**Affiliations:** Department of Chemistry, Lorestan University, Khoramabad 6815144316, Iran; kobrakhodarahmian@gmail.com

**Keywords:** chemotaxis, ionic liquid, drug delivery

## Abstract

Due to the growing prevalence of incurable diseases, such as cancer, worldwide, nowadays, the development of smart drug delivery systems is an inevitable necessity. Chemotaxis-driven movement of ionic liquid microdroplets containing therapeutic compounds is a well-known example of a smart drug delivery system. This review aims to classify, summarize, and compare ionic liquid-based chemotaxis systems in an easily understandable article. Chemotaxis is the basis of the movement of cells and microorganisms in biological environments, which is the cause of many vital biochemical and biological processes. This review attempts to summarize the available literature on single-component biomimetic and self-propelling microdroplet systems based on ionic liquids, which exhibit chemotaxis and spontaneously move in a determined direction by an external gradient, particularly a chemical change. It also aims to review artificial ionic liquid-based chemotaxis systems that can be used as drug carriers for medical purposes. The various ionic liquids used for this purpose are discussed, and different forms of chemical gradients and mechanisms that cause movement in microfluidic channels will be reviewed.

## 1. Introduction

Self-propelled systems based on micro/nanomotors are micro/nanoscale bioinspired motors that propel autonomously in a solution and convert chemical energy into movement. The ability to propel in response to an external stimulus is essential for many biological processes and life-forms. Specific cells, such as bacteria, motile cells, and other unicellular or multicellular organisms, exhibit tactic motions in response to chemical stimuli in their surrounding environment [1,2]. This tactical motion, so-called chemotaxis, plays an important role in many essential biological activities such as fleeing toxins, feeding, migration, and displacement of motile cells, as well as for reproductive cells [3], enzymes [4], and the immune systems [5,6].

Artificial biomimetic microdroplets (self-propelled microdroplet systems capable of moving in a microfluidic network) have been inspired by chemotactic organisms and developed by the placement of chemoattractants at the targeted destination within the microfluidic channels. Accordingly, a few mechanisms to propel microdroplets, including changeable wettability of a substrate surface by a chemical [7,8] electrochemical stimulus [9,10], temperature gradient [11,12], acoustic wave [13], magnetic field [14], and photostimulation [15], have been developed. Since most of these mechanisms comprise multi-component microdroplets and sophisticated experimental arrangements, they require applied external energy sources to move the microdroplets. Mechanism of spontaneous release of surfactants has been utilized to control the surface tension of aqueous systems to propel droplets autonomously on the aqueous–air interface. The surfactant interacts with the water molecules and reduce the surface tension of the aqueous system. Accordingly, by changing the surface tension of the fluid, it flows from regions with low surface tension towards regions with higher surface tension through a phenomenon so-called the Marangoni effect [16]. Marangoni flow is driven by a surface tension gradient at the interface of two fluids. Since the fluid with higher surface tension pulls its surrounding liquid more strongly than one with lower surface tension, the gradient in surface tension causes the fluid to flow towards regions of high surface tension. The surface tension gradient can be derived by using the temperature or concentration differences at the interface between the two fluids [17]. The direction of the Marangoni movement of a droplet can be controlled by manipulating the conditions under which the asymmetric release of the pre-loaded surfactant from the droplet occurs. Based on this fact, smart droplet systems were designed to move towards/away from a light source by using stimulant-responsive surfactants [18,19]. Self-propelled droplets were also used to solve complex mazes by exploiting surfactant releasing microdroplets chemotaxis through an aqueous solution with a strong pH gradient. The microdroplets opened up their ways through the maze to approach a region with different pH at the other end of the maze [20]. The great importance of this topic drove us to review ionic liquid (IL)-based chemotactic systems and their applications in drug delivery.

## 2. Ionic Liquids

### 2.1. Composition and Properties of ILs

**Figure 1:**
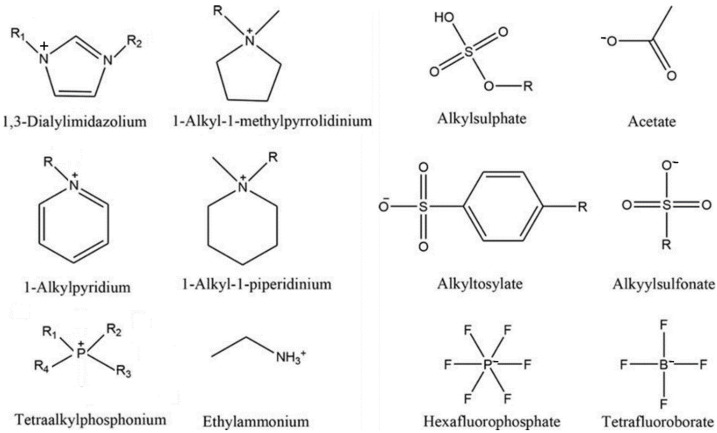
The most used cations and anions for the synthesized of ILs. Reproduced from [25] with permission.

Ionic liquids are typically defined as salts, compounds composed of ions, with melting points less than 100 °C. Indeed, they are salts with one or two large low-symmetry ions. They generally consist of large asymmetric organic cations and usually an inorganic anion. ILs made of asymmetric ions of various shapes and sizes have an irregular structure that let them liquify at low temperatures. Most ILs consist of organic cations and polyatomic anions, with large asymmetric structures, high diffuse charges, and weak inter-ionic interactions. The large size of their molecules and the nature of the chemical groups of the anions cause the charges to be scattered over their constituent ions. The reduced electrostatic forces between the anion and cation and their asymmetry prevent the formation of a stable crystalline structure, and as a result, ILs are liquid at low temperatures (below 100 °C) [21,22]. Therefore, in any IL, at least one ion has a delocalized charge, and an organic component is required to prevent the formation of a stable crystal lattice. The specific ions used in ILs show moderate polarization charge densities due to the delocalization of the molecular charge. In many ions that make up ILs, such as pyridinium ([C_5_H_6_N]^+^) and imidazolium ([C_3_H_5_N_2_]^+^), the charge is delocalized through conjugation and hyperconjugation. In some symmetrical ions involved in IL formation, such as phosphonium ([PH_4_]^+^) and ammonium ([NH_4_]^+^) cations or hexafluorophosphate ([PF_6_]^−^) and tetrafluoroborate ([BF_4_]^−^) anions, the charge is delocalized over their entire structure [23]. In recent years, ILs have been widely applied as green solvents due to their extraordinary properties, such as high density (higher than water), large viscosity (much more than molecular solvents), very low vapor pressure (non-volatility), non-flammability, high thermal stability, low chemical reactivity, high ionic conductivity, sizeable electrochemical window, extensive liquidus range, and ability to solvate compounds of widely varying polarity, as well as prominent solvation capability for organometallic, inorganic, and organic compounds. These features have provided a wide range of applications for ILs [21,24]. Some of the cations and anions that are commonly used in ILs structure are shown in Figure 1 [25]. Names and abbreviations of some common ILs are presented in Table 1 [26].

The preparation of ILs in high quantities does not cause remarkable difficulties. They can be synthesized with different properties by altering the composition of anions and cations. The three primary procedures for the preparation of ILs are as follows: (i) direct neutralization of the acid with a proper base; (ii) metathesis preparation of ILs in appropriate solvents; and (iii) solvent-free anion metathesis by melting or grinding [27]. When excess acid or base is used, the quantities of reactants can be relatively non-stoichiometric or stoichiometric, leading to complete or partial ionization of the acids and bases [28]. The acid-base reactions that do not produce by-products are quite exothermic reactions and therefore may be more challenging to scale up. However, the commonly used metathesis reactions can directly contaminate ILs with halide anions, which makes further purification needed.

### 2.2. Classification of ILs

ILs are generally categorized into two groups. The first group has unique adjustable intrinsic physical and chemical properties, such as density, viscosity, conductivity, solubility, and high thermal and chemical stability. These ILs, so-called room-temperature ionic liquids (RTILs), are free-flowing liquids at room temperature and are considered greener alternatives to volatile organic solvents (VOSs) due to their negligible vapor pressures. Mixtures of aluminum chloride and *N*-butyl pyridinium chloride in different molar ratios (tetrachloroaluminate) can be mentioned as a well-known example of this group. The specific targeted behavior of the second group of ILs (e.g., dialkyl imidazolium with neutral weakly coordinating anions, such as [BF_4_]^−^ and [PF_6_]^−^), offered the potential to regulate some of these physical and chemical properties, allowing the formation of task-specific ILs (TSILs) [29]. The functional groups of TSILs are covalently linked to the cation, anion, or both. As a result, their potential applications are far beyond those of conventional ILs. These ILs can be combined in solutions, and so, the synthesis supported in a homogeneous solution can be obtained, which is a significant advantage. Some TSILs comprise active pharmaceutical ingredients (API) and are exploited to obtain specific desirable biological properties.

ILs are also classified based on the nature of their constituent ions. Accordingly, ILs are divided into two main categories: cationic ILs and anionic ILs. Cationic ILs include bulky organic cations such as pyrrolidinium, guanidinium, morpholinium, sulfonium, ammonium, phosphonium, pyridinium, and imidazolium’s derivatives, while anionic ILs contain bulky organic anions (such as methanesulfonate, alkylsulfate, tosylate, trifluoroacetate) or bulky inorganic anions (such as [PF6]^−^, [BF4]^−^, [Cl]^−^, [Br]^−^, and TFSI or bis(trifluoromethane)sulfonimide) [30]. Conventional ILs are composed of monocation and an anion. Dicationic ILS (DILs) have been recently introduced as a new category and attracted great attention due to their interesting features. They are classified as homoanionic DILs and heteroanionic DILs. They can be further divided into symmetrical and asymmetrical DILs for both homoanionic and heteroanionic classes [31].

### 2.3. ILs as Promising Alternatives to Traditional Solvents

The use of ILs as environmentally friendly solvents for many catalytic and synthetic processes is one of the goals of green chemistry to provide a cleaner and more sustainable chemistry. ILs are green solvents that can dissolve a wide spectrum of inorganic, organic, and organometallic compounds. Since organic salts are liquid at room temperature, they can be used as solvents for many different reactions, including catalytic reactions. Additionally, they have very low vapor pressures and are relatively thermally stable, as well as their possibility of evaporation during chemical or physical processes is insignificant. The latter feature prevents environmental and protective issues due to volatilization, as it is a serious issue for conventional organic solvents. Accordingly, in a rising trend, ILs are used as novel solvent systems in organic synthesis, solvent extraction, separation, and electrochemistry, instead of traditional solvents that are generally volatile, flammable, and toxic [32].

One of the applications of ILs is the extraction of heavy metal ions due to the hydrophobicity and water immiscibility of specific ILs, which allow their use in solvent extraction of hydrophobic complexes of heavy metal ions. In a study, a room-temperature ionic liquid, 1-butyl-3-methylimidazolium hexafluorophosphate [C_4_mim][PF_6_], was used as an alternative solvent for the extraction and separation of Cu^2+^, Pb^2+^, Zn^2+^, Cd^2+^, and Hg^2+^ in aqueous solutions. To improve the distribution ratios, dithizone was used as a chelating agent to form neutral lipophilic metal-dithizone complexes to enable the extraction of the metal ions by using [C_4_mim][PF_6_] [33]. Because of the intense chromogenic properties of dithizone, it allowed using UV–Vis spectrophotometric determination of the studied metal ions [34]. The extraction results demonstrated high distribution ratios of the metal complexes between [C_4_mim][PF_6_] and the aqueous sample. In another study, different functionalized ILs were prepared by combining thiourea, thioether, and urea with imidazolium cations, derivatized by incorporating chelating groups, to induce task-specific functionality. The functionalized TSILs were exploited as the extractant for the solvent extraction of Hg^2+^ and Cd^2+^ ions. Mixing these TSILs with [C_4_mim][PF_6_] resulted in significant improvements in the distribution ratios [35]. Some of the most important published articles in using ILs as green solvents, microreactors, and extractants are summarized in Table 2.

### 2.4. Cytotoxicity of ILs

In recent years, the use of ILs as benign solvent systems in pharmaceutical and medical applications has increased dramatically. However, the use of ILs as green solvents in medicine and biomedicine is still debatable. Many researchers are concerned about the toxicity of ILs as the most critical challenge for their biological applications in drug delivery, which is a serious issue in this area due to the lack of scientific knowledge on it. However, for pharmaceutical applications, non-toxic ILs can be synthesized by tailoring their physical and chemical properties by changing their anion/cation combinations [45]. Accordingly, there have been recent reports in which non-toxic ILs were produced by combing inorganic anions and biocompatible organic cations [46,47]. In addition, biocompatible ILs can be synthesized by changing the properties of the side chains appended to the IL cations. For instance, Morrissey et al. demonstrated that the toxicity of ILs was significantly reduced by incorporating ‘ether’ groups with the ester side-chains compared with ‘alkyl’ ester derivatives [48]. It should not be forgotten that many useful and widely used chemicals in pharmaceutical preparations are toxic. For example, many pharmaceutical excipients, such as nonionic surfactants (e.g., polysorbate 80) and dimethyl sulfoxide (DMSO), display similar toxicities to ILs. DMSO, a well-known chemical enhancer for pharmaceutical formulations, is reported to be safe up to 10%, while its toxicity significantly increases at higher concentrations [49,50]. Polysorbate 80 has been demonstrated to be nonmutagenic in micronucleus tests. It was also shown to be noncarcinogenic in laboratory animals, while some studies have shown that it enhances the activity of known chemical carcinogens. Polysorbates have also shown minor human skin irritation and sensitization effects [51].

Generally, ILs with hydrophilic anions and a short alkyl chain attached to imidazolium cation show low toxicities [52]. More interestingly, IL solvents exhibit good biodegradability and low toxicity for drug delivery. As a result, the toxicity of ILs is not an obstacle to using them as a solvent or ingredient in pharmaceutical preparations and drug delivery.

## 3. Self-Propelled Microdroplets

In non-equilibrium conditions, liquid droplets can become active systems by coupling to their surrounding environment through a steady flow of energy or matter, capable of mimicking various life-like functions. They are propelled in response to external fields, such as magnetic, electric, light, and acoustic waves, temperature, and concentration gradients, as well as chemical reactions. Though in some cases, these phenomena can lead to very sophisticated physicochemical behaviors; however, they are generally based on surface-tension effects and relatively simple surface chemistries. Herein, first, an overview of some of these phenomena, such as magnetotaxis, acoustic waves, and phototactic, is provided, and following that, the phenomenon of chemotaxis is reviewed in detail. Among the mentioned phenomena, chemotaxis is more like the movement of biological microswimmers and living cells, and so, it is more promising to develop efficient and applicable drug delivery systems.

**Figure 2:**
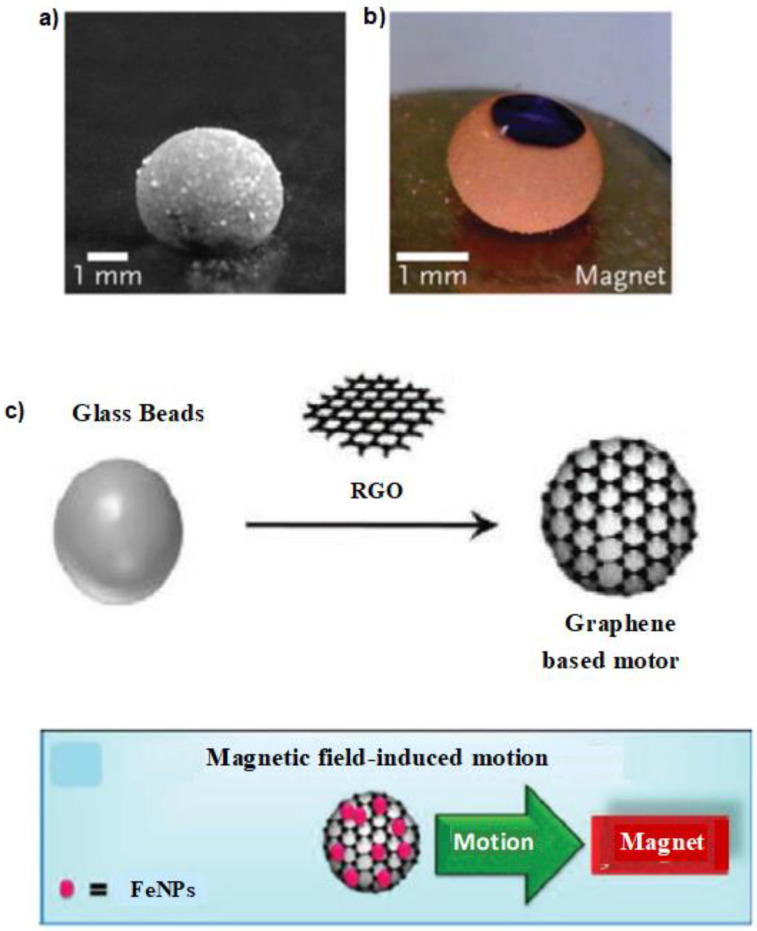
Formation of marble-like liquid microdroplets using (**a**) Fe_2_O_3_ and (**b**) Fe_3_O_4_ nanoparticles after suspension in a liquid. The marble can be opened and exposed to it water content (dark blue circle) when the magnetic nanoparticles are pulled by the magnetic field. (**c**) Mechanism of preparation and motion of reduced graphene-oxide motor. Reproduced from [14] with permission.

Droplets containing magnetic additives, such as porous silicon microparticles infused with magnetite, can be manipulated by magnetic fields. However, such systems require to be carefully adjusted to prevent the magnetic particles from escaping around the droplet, and the field must be strong enough to facilitate the droplet movement. Dorvee et al. studied one-dimensional photonic crystals made from porous silicon microparticles. The porous microparticles were converted to amphiphilic constructs via oxidation and then added to a two-phase liquid, such as water-dichloromethane. These microparticles spontaneously aligned and accumulated at the interface of the two phases. When superparamagnetic magnetite nanoparticles were incorporated into the porous nanostructure of the microparticles, they could have chased liquid microdroplets in an external magnetic field. The microdroplets were identified using a peak that appeared in the optical reflectivity spectrum of the photonic crystal [53]. In similar systems using Fe_2_O_3_ [54] and Fe_3_O_4_ [14] nanoparticles, marble-like microparticles were formed when the microparticles were suspended in a liquid. When a magnetic field was applied to the system, the magnetic microparticles were pulled by the field. The marble-like microparticles opened under the effect of the magnetic field and exposes their water contents, such as those shown in Figure 2 [14,54,55].

**Figure 3:**
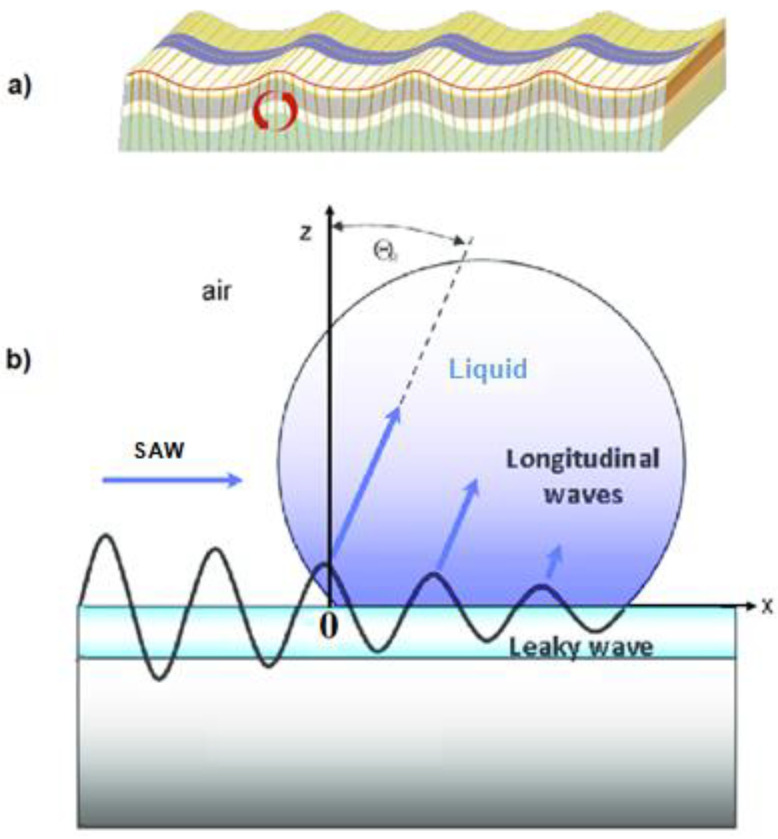
(**a**) Depiction of a SAW-propagating liquid on a piezoelectric substrate and (**b**) schematic illustration of the interaction between a SAW (which is propagating from left to right, and at x = 0, hits the liquid) and a liquid microdroplet on the surface of the piezoelectric substrate. A longitudinal sound wave is irradiated into the fluid under a refraction angular of Θ. Reproduced from [13] with permission.

In a different study, the interaction of surface acoustic waves (SAW) with a liquid on the surface of a piezoelectric substrate led to an increase in acoustic radiation pressure in the fluid. This pressure caused an internal stream of SAW-mediated in the fluid and the actuation of small droplets. A snapshot of a SAW-propagating liquid on a solid surface and the origin of such acoustically induced internal streaming is shown in Figure 3 [13].

**Figure 4:**
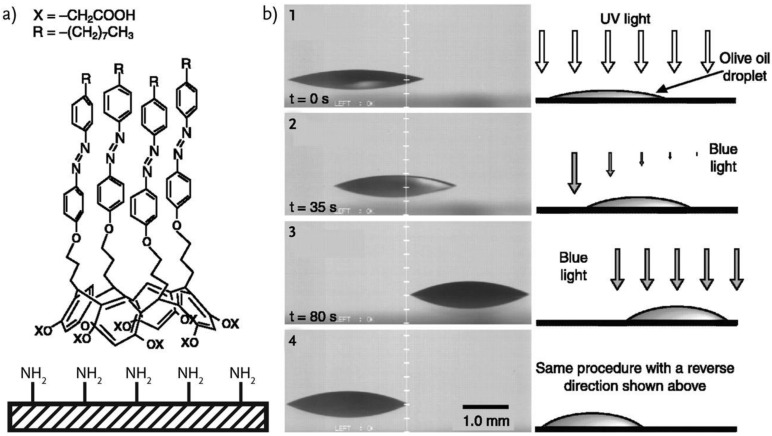
(**a**) Monolayer formed by azobenzene-containing CRA-CM amphiphiles and (**b**) sequence of light-driven motion of an olive oil droplet on the CRA-CM photoresponsive surface. The olive oil droplet on a *cis*-rich surface moves in a direction of a higher-energy surface by asymmetric irradiation with 436 nm light perpendicular to the surface. The contact angles were changed from 18° (1) to 25° (3). The droplet movement route was controllable by varying the photoirradiation direction. Reproduced from [55] with permission.

The manipulation of the macroscopic movement of liquid microdroplets on the smooth surface of a solid is also possible using photo-irradiation of a photoisomerizable monolayer that covers the surface. When a liquid microdroplet is placed on a modified substrate surface with a derivatized Calix[4]resorcinarene with azobenzene photochromic units, asymmetric photo-irradiation causes a gradient in free energy of the surface due to the photoisomerization of the superficial azobenzenes, resulting in the directional movement of the droplet [55]. Figure 4 shows the directional movement of a droplet on a silica plate modified with a o-carboxymethylated Calix[4]resorcinarene (CRA-CM) photoresponsive surface layer when the surface is exposed to asymmetrical irradiation with blue light.

## 4. IL-Based Chemotaxis

Chemotaxis, the movement of droplets in response to external chemical stimuli, is inspired by the similarities of motile cells and bacteria that represent tactical motions in response to biochemical stimuli [2]. When used in chemical simple reactions, chemotactic droplets can move and perform intelligent functions, such as solving maze and drug delivery systems. Chemotactic droplets can maintain primary forms of metabolism and self-replication and indicate evolutionary alterations, such as biological cells, by using more sophisticated chemicals.

**Figure 5:**
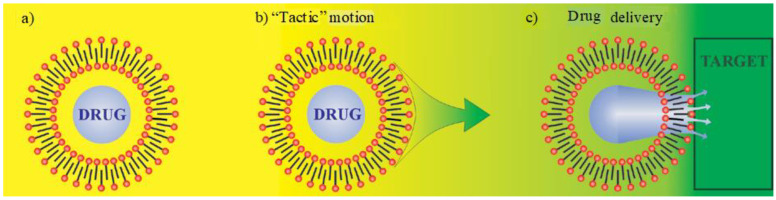
Drug delivery by an artificial tactic motion: (**a**) Artificial cell, a microdroplet stabilized by a bilayer of a fatty acid or phospholipid molecules, containing a drug; (**b**) controlled tactic motion by the Marangoni effect to the target; and (**c**) drug delivery from the artificial cell to the target through the membrane of the target cell. Reproduced from [63] with permission.

The application of chemotaxis for drug delivery to cancer cells is justified by the fact that cancer cells have a lower pH compared to the normal tissues in the human body [56]. Because of their higher metabolism rates, cancer cells have higher resting temperatures than normal cells [57]. Since the human body’s living cells are composed of a self-assembled phospholipid bilayer, a stabilized compartment of a layer of self-assembled fatty acid molecules is usually regarded as a prototype of living cells. Therefore, the use of fatty acid chemistry to design an autonomous moving shell is a promising choice in chemotaxis. An artificial cell system can consist of a micellar vesicle or a stabilized oil droplet with a phospholipid or fatty acid mono or bilayer, which is able to move and respond to environmental stimuli. It can accommodate drugs or pharmaceutical carriers such as nanoparticles. The membrane of this small compartment protects the drug from leakage. Autonomic motion (taxis) of the synthetic cell can be controlled by chemical asymmetrical reactions at the upper and lower edges of the cell membrane [58] or the Marangoni effect. The protonation rate of the fatty acid molecules in a membrane varies by chemical gradient and consequently results in a gradient of surface tension in the membrane. This can sustain an autonomic tactical movement of stabilized fatty acid compartments [59,60]. The most important aspect of the tactical motion of these types of artificial cells is drug delivery. Such a tactical small droplet can carry drugs to a specific position, where the drug must be released (Figure 5). The release can be inactive by diffusion through the membrane or active by specific donor-acceptor reactions between the target surface and the cell [61,62,63].

**Figure 6:**
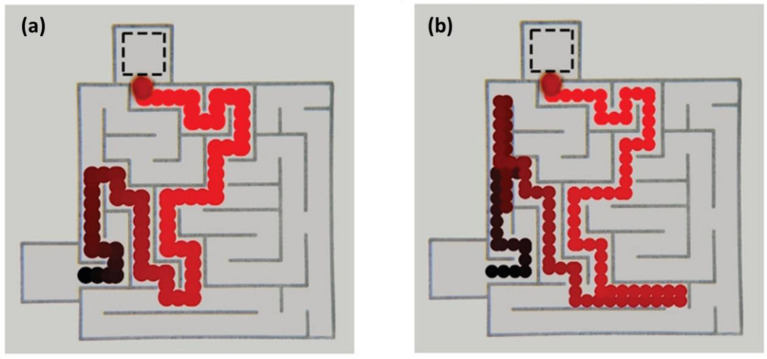
Maze solving by chemotactic droplets (HDA in DCM) in a pH gradient in a maze (the droplets move toward regions of low pH through the shortest possible path). (**a**) The HDA/DCM droplet solves the maze without any deviation, and (**b**) the droplet drifts in two locations but eventually corrects itself to find the shortest path leading to the maze’s exit. Reproduced from [20] with permission.

**Figure 7:**
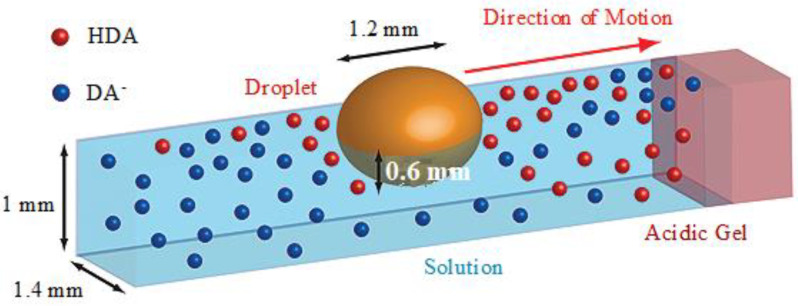
Non-uniform distribution of HDA at the liquid–air interface, resulting in variations of surface tension over the droplet surface. Due to the presence of more HDA in the direction of the acid source (low pH), the forces and flows are asymmetric. Reproduced from [20] with permission.

In this regard, Istvan Lagzi et al. investigated a system consisting of small droplets based on the combination of acid/base chemistry and surface tension gradients. This self-propelled chemical construct, comprising a droplet of a water-immiscible organic solvent (dichloromethane, DCM) containing hexyldecanoic acid (HDA), can solve mazes in response to chemical stimuli, as shown in Figure 6 [20]. When exposed to the pH gradient within the maze, the droplet moves toward regions of low pH and finds the shortest possible paths. The results showed that when the gradient of pH was uniform throughout the maze or when the droplet lacked HDA, no directional movement of the droplet was observed. In this system, the presence of both HDA and a gradient of pH is essential for the droplet’s chemotaxis. Solving the maze with HDA-containing droplets is achieved by surface tension gradients resulting from the non-uniform distribution of HDA on the liquid–air interface, as depicted in Figure 7 [20]. The diffusion of HDA from the droplet causes its deprotonation in the pH gradient, while there is more protonated HDA in the direction of the acid source, i.e., in lower pH regions. This asymmetrical distribution results in a surface tension gradient by the concentration of protonated HDA mainly characterized at the liquid-air interface [64]. Eventually, this gradient of surface tension leads to a convective flow, such as the Marangoni flow, which is responsible for the motion of the microdroplet.

**Figure 8:**
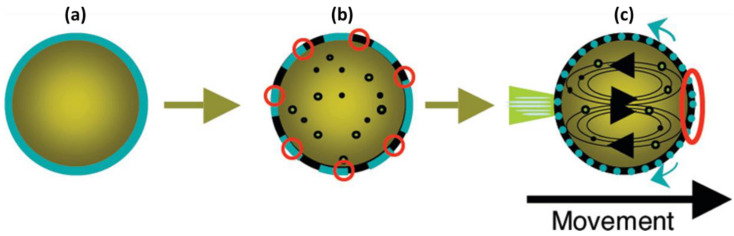
Schematic representation of the stages of events resulting in the appearance of surface motion and flows: (**a**) A symmetrical oil microdroplet immersed in the aqueous phase; (**b**) the oil microdroplet covered with surfactant; and (**c**) internal discrete structures within the oil microdroplets cause oscillatory movements and expose the oil to the alkaline external solution (possible sites of hydrolysis are shown by red circles). After the formation of the vortices, the convection flow begin (black arrows inside the oil phase), the surfactant moves to the prior pole, and the hydrolysis of the oil microdroplet is localized (red circle). The blue lines indicate the exit of the surfactant from the interface, which causes the microdroplet to move. Reproduced from [59] with permission.

Hanczyc et al. developed another simple system of moving single droplets based on fatty acid chemistry. This system is comprised of oleate surfactant (oleic acid in alkaline aqueous solution, pH = 11) and an oil microdroplet (anhydride oleic acid in nitrobenzene). The surface of the oil microdroplet was coated by the surfactant after immersing it in the surfactant medium, but no movement was seen in the first moments (Figure 8a). Subsequently, the surface symmetry was broken, and distinct structures formed within the oil microdroplet. These structures exposed the oil to the alkaline medium by spontaneous oscillatory movements and enabled the hydrolysis of anhydride oleic to oleate. The hydrolysis reaction lowered the pH near the droplet’s surface, which was under the influence of surface tension (Figure 8b). This combination of pH gradient and changes in the surface tension resulted in the movement of the microdroplet by the formation of two counter-rotating vortices within the droplet (Figure 8c) [59].

**Figure 9:**
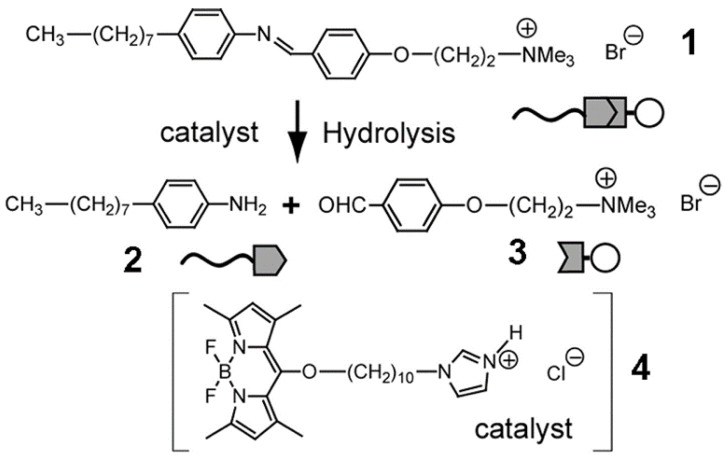
Schematic illustration of the hydrolytic reaction in an oil self-propelled droplet. Chemical fuel (surfactant, **1**) produces aniline lipophilic derivative (**2**) and benzaldehyde hydrophilic derivative (**3**) in the presence of the catalyst (**4**). Reproduced from [60] with permission.

In another chemotactic system, a distinctive surfactant called [[(trimethylammonio) ethoxy]-benzylidene-octylaniline bromide] ([[(TMA) OEt]-B-OA Br]) was combined with octylaniline (OA) and (4-formylphenoxy) ethyltrimethylammonium bromide ((4-FP) TMEAB) and used as a chemical fuel to power microdroplet motion. A fluorescent catalyst was utilized to increase the hydrolysis of the surfactant to its benzaldehyde and aniline derivatives, as depicted in Figure 9 [60].

**Figure 10:**
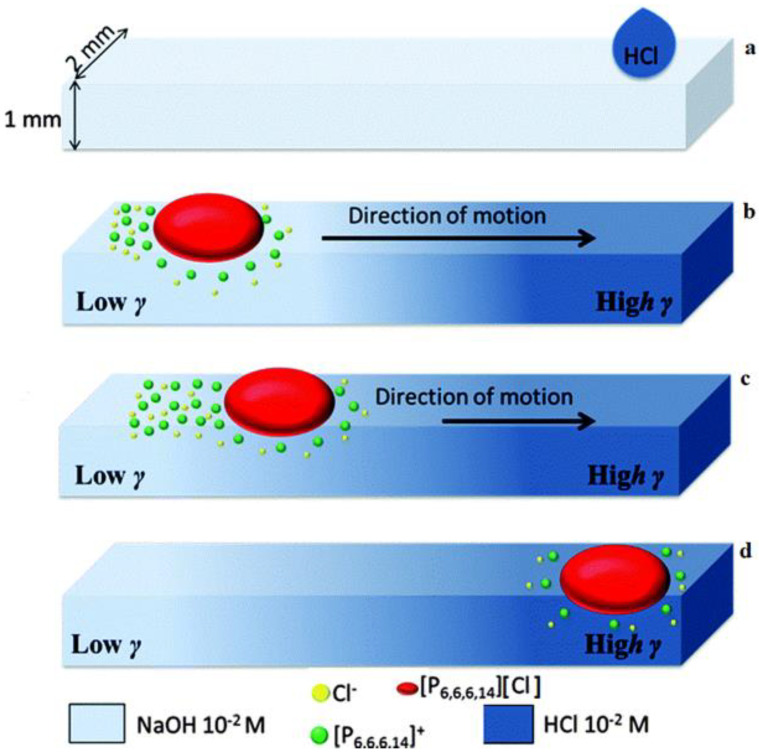
Motion of an IL droplet in an open channel fluidic system: (**a**) Creating a concentration gradient of Cl^−^ by adding hydrochloric acid (10^−2^ M) in the fluidic channel initially filled with a NaOH solution (10^−2^ M); (**b**) an IL droplet is placed at the beginning of the channel that begins to release the surfactant into the solution and creates a surface tension gradient around the droplet; (**c**) the droplet then moves towards regions with the highest surface tension; and (**d**) the droplet arrives at the favorite destination. Reproduced from [65] with permission.

Francis et al. developed another system of chemotactic spontaneous motion based on IL droplets at the aqueous–air interface. They used IL droplets of trihexyl(tetradecyl)phosphonium chloride ([P_6_,_6_,_6_,_14_][Cl]). The spontaneous motion of these droplets along the aqueous–air interface by chemical gradient is due to the asymmetric release of the cationic surfactant, [P_6_,_6_,_6_,_14_]^+^, from the droplet into the aqueous phase. When a surfactant (as a long-chain amphiphile with a hydrophobic tail and hydrophilic head) is released from a hydrophobic microdroplet at the aqueous–air interface of a solution, it interacts with polar molecules present at the solution surface and interrupts their attraction forces. Accordingly, a flow is created in the bulk solution, and the fluid flows from regions with low surface tension to high surface tension, which is considered the Marangoni flow. When a droplet of ([P_6_,_6_,_6_,_14_][Cl]) floats in an aqueous medium, [P_6_,_6_,_6_,_14_]^+^ ions diffuse from the droplet into the solution, interact with the superficial molecules, and decrease the surface tension (Figure 10). At the same time, a proportionate amount of the Cl^−^ ion is lost to maintain the charge neutrality within the droplet. As a result, the release of the surfactant is dependent on the solubility of the Cl^−^ ion. Therefore, the rate of release of the opposite ion, [P_6_,_6_,_6_,_14_]^+^, is dependent on the local concentration of Cl^−^ in the aqueous solution. Accordingly, when the IL droplet was immersed in an aqueous solution with a concentration gradient of Cl^−^, the asymmetric release of the [P_6_,_6_,_6_,_14_]^+^ ion resulted in a surface tension gradient around the droplet. Accordingly, Marangoni-like flows were created in the solution and caused the droplets to move from regions with lower surface tension to regions with higher surface tension (higher concentration of Cl^−^ in the solution).

Since the partitioning of the low solubility cationic surfactant in the aqueous media is dependent on the solubility of its counter ion, the droplet motion can be controlled by any concentration gradient in the solution that affects the solubility of the anion. Therefore, the chemotactic movement of the IL droplet is not limited to concentration gradients of its counter anion, but it can also be controlled by concentration gradients of other anions, such as bromide, by adding KBr or sulphate from Na_2_SO_4_ [65].

**Figure 11:**
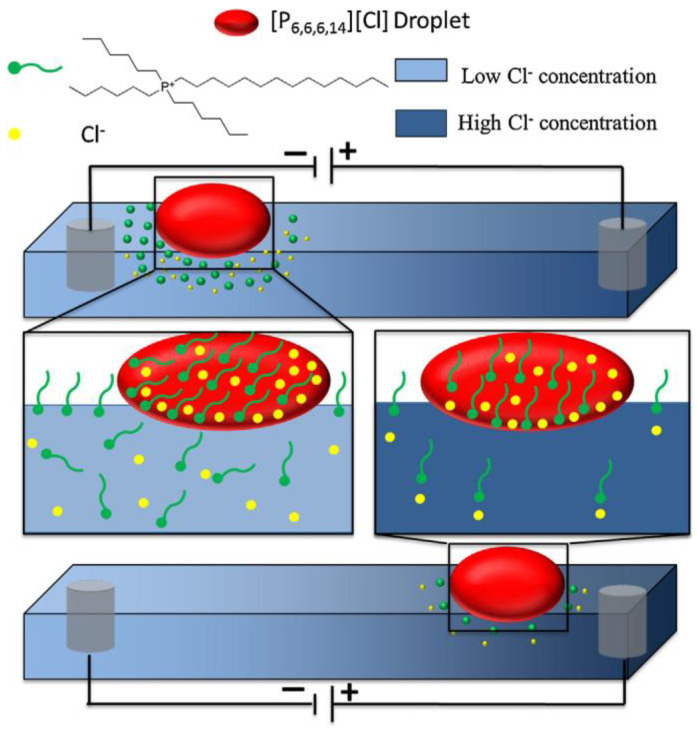
Relative solubility of the surfactant droplet, [P_6_,_6_,_6_,_14_][Cl], in lower (at the cathode, **top**) and higher (at the anode, **bottom**) concentrations of Cl^−^. Reproduced from [66] with permission.

Francis et al. demonstrated that when a single component of IL droplets can respond to an external electrical stimulus, it shows electrotactic motion. They inserted three-dimensional printed (3DP) mesh electrodes into a fluidic channel filled with a NaCl (10^−3^ M) solution to produce the required ion gradient. Electrotactic actuation of a [P_6_,_6_,_6_,_14_][Cl] droplet at the liquid–air interface of the solution was achieved by applying an electrical external field through the fluidic channel. This caused the electrolyte ions to migrate to the corresponding counter electrodes and created a concentration gradient of Cl^−^ across the channel. Then, the potential was switched ON, and the [P_6_,_6_,_6_,_14_][Cl] drop floated on the surface of the solution on the cathode side. The concentration gradient of Cl^−^ led to the asymmetrical release of the cation, [P_6_,_6_,_6_,_14_]^+^, and moved the droplet to the anode, as depicted in (Figure 11) [66]. By reversing the polarity of the electrodes, the concentration gradient and motion of the droplet could simply be reversed. A significant advantage of the electrotactic motion of droplets over the chemotactic motion is the ability of concentration gradients to be established and varied dynamically and retained for longer periods of time. These features lead to very flexible control of the direction and speed of the IL droplet’s motion.

## 5. Smart Drug Delivery Using ILs

In the pharmaceutical industry, active pharmaceutical solids (APIs) are divided into two main groups: single-component and multi-component APIs. Single-component APIs are available as singular compounds, while multi-component APIs can be available as common solvates/hydrates (APIs + solvent/water), co-crystals (APIs + crystal co-former), and salts (APIs + counterion) [27,67]. Solid APIs can exhibit polymorphism as a crystallized compound in more than one crystalline form [68]. Due to differences in the physicochemical properties of various polymorphs, their bioavailability, absorption, and solubility can be significantly different. These differences lead to out-of-solution crystallization of the pharmaceutical and thus adversely affect their bioavailability [27].

Another common problem with solid APIs is particle size. Essential factors related to drugs, such as penetrability, dissolution rate, uniform distribution, and suspendability, can be improved by decreasing or increasing particle size. Therefore, particle size is a critical factor that needs serious attention to produce solid APIs and control polymorphism, product solubility, and crystal properties. Over the past two decades, ILs have been widely utilized as green solvents in a variety of applications, including drug formulations and drug delivery [69]. Extraordinary features of ILs have made them useful solvents/carriers in biomedical applications and pharmaceutical applications as an essential component of drug formulations. Such formulations may include the formation of API-IL conjugates [23] or IL-containing solids in oil microemulsions/nanodispersions [70,71] for topical/transdermal drug delivery purposes.

Most drugs are highly hydrophobic and insoluble or sparingly soluble in water. These drugs are never formulated directly due to the poor solubility and problems with their delivery. Accordingly, some procedures have been developed to conquest this problem to increase the drug solubility by utilizing excipients, such as dimethyl sulfoxide (DMSO) and ethanol. However, the use of these excipients in high concentrations, in addition to toxicity, will lead to undesirable side effects [72]. ILs have recently emerged as a promising alternative to these excipients and overcome the limitations. Hereupon, they have been used to develop IL-based nanoemulsions with significantly improved solubility [73]. A nanoemulsion or nano-sized emulsion is a thermodynamically stable isotropic mixture of two immiscible liquids that form a single phase using a surfactant or co-surfactant as an emulsifying agent [74]. The microemulsion carrier systems can be water-based or oil-based depending on the polarity of ILs [75]. Nanoemulsions have remarkable features, including extensive surface area, droplet small size, and long-term physical stability against creaming or sedimentation [76,77,78]. Due to these excellent properties, nanoemulsions have been exploited as promising carriers for drug delivery, particularly in the transdermal delivery of different drugs [79,80]. They can maintain sustain control over the delivery of active portions and protect the bioactive compound [78]. Therefore, drug delivery using nanoemulsions is considered a situated method to conquest the side effects in the therapy of diseases. The published articles in the field of drug delivery, in which ILs play a significant role in the dissolution and stabilization of the drugs, as well as the drug delivery reports that have used chemotactic systems based on self-propelled IL droplets, are summarized in Table 3.

Dalvand et al. reported an in vitro smart delivery and release system for epirubicin anticancer drug using a moving IL microdroplet, [P_6_,_6_,_6_,_14_][Cl], through a 3D printed microfluidic device. The movement of the IL microdroplet was reversibly controlled by an external electrical field at a low voltage. The asymmetrical permeation of the [P_6_,_6_,_6_,_14_]^+^ cation from the microdroplet into an aqueous medium, due to the concentration gradient of chloride, resulted in the motion of the microdroplet. The 3D printed microfluidic device at the end of the channel was equipped with a suitable receptor and consisted of a hollow-fiber filled with phosphate buffer. After passing through the microfluidic channel by microdroplet motion, the drug was gradually released into the receptor buffer solution. The receptor solution was then injected into an HPLC system to quantify the released drug.

The results of a study by Moniruzzaman et al. showed that an IL in an oil microemulsion can be used to increase the solubility and transdermal delivery of acyclovir (ACV). Dimethylimidazolium dimethylphosphate [C_1_mim][(CH_3_O)_2_PO_2_] was used as the IL, and a blend of polyoxyethylene sorbitan monooleate (Tween-80), sorbitan laurate (Span-20), and isopropyl myristate (IPM) was applied as the oil phase [72]. This system provided a high solubility for ACV in the IL phase, and an excellent stability and low cytotoxicity was observed for the IL-based carrier. However, the unknown biocompatibility of this formulation was reported as a limiting factor.

In another study, IL-in-oil nanoemulsions were successfully formulated using two types of ILs, 1-butyl-3-methylimidazolium hexafluorophosphate [Bmim][PF6] and 1-hexyl-3-methylimidazolium chloride [Hmim][Cl], in Tween-80/Span-20 for the drug delivery of piroxicam [83]. Both ILs showed proper solubility for the drug by encapsulating it into the nanoemulsion micelles.

Holler et al. used a chemotactic 1-decalol droplet for the transport of live cells under sterile conditions [85]. 1-Decanol droplets in an aqueous solution of decanoate at a high pH (pH 11–12) become chemotactic in the presence of an external chemical gradient. Such droplets were used as the carrier for living cells. A hydrophobic alginate capsule, as a protective unit, was loaded into the droplets and carried along through an external chemical gradient (chloride), created by adding sodium chloride (3 M NaCl). When the droplet with alginate capsule cargo reached the target destination, the connection of the droplet and alginate capsule was disrupted, and the cargo was deposited.

Joseph et al. reported an artificial nanoscopic organic system with a chemotaxis-driven movement created by glucose enzymatic conversion. Glucose oxidase alone or in combination with catalase was encapsulated into biocompatible nanoscopic asymmetric polymer vesicles. The vesicles were self-propelled in response to a glucose concentration gradient and moved toward the high-concentration regions. Poly[(2-methacryloyl)ethylphosphorylcholine]-poly[2-(diisopropylamino) ethylmethacrylate] (PMPC-PDPA) and poly[oligo(ethyleneglycol) methylmethacrylate] (POEGMA-PDPA) were exploited as the asymmetric polymersomes [82]. They showed high flexibility for different combinations of substrate/enzyme. The combination of glucose oxidase and catalase provided an efficient chemotaxis polymersome system in the presence of the glucose external gradient. However, the permeability of the polymersome membrane was shown to be a limiting factor.

In another research, an effective formulation for oral insulin was investigated by using choline and geranate (CAGE) ionic liquid. CAGE considerably enhanced the insulin paracellular transport while protecting it from the enzymatic decomposition and due to the interaction with the mucosal layer [84]. Although the gastrointestinal tract is known as an important barrier to oral delivery of biological substances, this proposed IL-formulation significantly enhanced the absorption of oral insulin. Additionally, the proposed formulation showed high biocompatibility and good stability.

Bielas et al. reported a new type of carrier for the delivery of biologically active pharmaceutical anions (attached to silicates, Sal) based on grafted copolymers of a poly-ionic liquid [86]. For this purpose, a Sal-containing IL/methacrylate monomer was copolymerized with methyl methacrylate (MAA) using a multifunctional macroinitiator through a controlled radical polymerization route. The results were evaluated by changing the degree of grafting and composition of the side chains (ratios of ionic/nonionic units). The graft copolymers were self-assembled into spherical nanostructures with sizes up to 73 nm in aqueous solutions. Then, the prepared graft copolymers were evaluated for anti-inflammatory (for IL-6 and IL-8 proinflammatory cytokines) and antibacterial activities (towards *E. coli*), as well as for the estimation of cytotoxicity towards human cells (BEAS-2B and NHDF). The results demonstrated very low toxicity against the studied human cells, confirming the possible application of these poly(ionic liquid) nanocarriers as novel drug delivery systems.

Although ionic liquids as green and biodegradable solvents have attracted considerable attention for drug delivery and drug formulation applications, little is known about their biocompatibility and toxicity [88,89]. On the other hand, some recent studies on the toxicity of ILs questioned their safety [90]. In particular, the biocompatibility of ILs in the human body has not been conclusively proven [91]. Therefore, it is too early to perform clinical trials using IL-based drug delivery systems. In fact, much more studies are needed to prepare and test completely safe ILs for in vivo and clinical applications.

## 6. Conclusions and Future Trends

Stimuli-responsive materials have been recently demonstrated to be novel devices for many applications, including programmed cargo delivery in microfluidic systems and self-propelled micro-reactors capable of performing small-scale reactions at intended destinations. This is useful for realizing low-cost autonomous chemical carriers and analyzers that can perform sophisticated microfluidic processes by incorporating actuator-controlled synthetic droplets in microfluidic devices. Chemotaxis based on IL droplets is a well-established method among these systems that provides the ability of movement to self-propelled IL droplets in response to an external chemical stimulus to achieve a target, specific position. Chemotactic systems play an essential role in the movement of biological microswimmers and living cells in biological processes. Accordingly, chemotaxis has been exploited to mimic biological microswimmers and microreactors, particularly for drug delivery purposes. The most investigated systems are self-propelled droplets that spontaneously move in a determined direction by a chemical external gradient. The drugs are pre-loaded into these moving droplets to be delivered to the desired positions. The autonomous motion of the droplets does not need any energy external source because it is driven by a Marangoni flow, which is caused by a self-sustained interfacial tension gradient during the solubilization of the droplets’ constituents in a micellar surfactant solution. Depending on the purpose and planning, chemotactic systems can be designed that perform a specific activity to reduce the risk of malfunction. Such systems designed for drug delivery applications can significantly reduce the side effects of possible treatments.

IL-based micro- and nanoemulsions have been proven as promising systems for drug formulations and delivery of sparingly soluble drugs that are difficult to administer in other ways. These systems have extraordinary chemical and physical formulation stability as well as very low toxicity. However, the biocompatibility of these systems is almost a limiting factor and needs further investigation. Accordingly, the greatest efforts should be devoted to developing new biocompatible and safe IL-based drug carriers with excellent properties for specific applications to increase the remedial influence and decrease the side effects of drugs.

## Figures and Tables

**Table 1:** Full names and abbreviations of some commonly used ILs. Reproduced from [26] with permission.

Name	Abbreviation
triisobutyl (methyl) phosphonium tosylate	[P_1,4,4,4_][Tos]
tetrabutylphosphonium dicyanamide	[P_4,4,4,4_][DCA]
trihexyltetradecyl phosphonium dicyanamide	[P_6,6,6,14_][DCA]
trihexyltetradecyl phosphonium bis (trifluoromethanesulfonyl) imide	[P_6,6,6,14_][Ntf_2_]
trihexyltetradecyl phosphonium dodecylbenzenesulfonate	[P_6,6,6,14_][DBSA]
trihexyltetradecyl phosphonium chloride	[P_6,6,6,14_][Cl]
1-methyl-3-octylimidazolium tetrafluoroborate	[OMIM][BF_4_]
1-ethyl-3-methyliidazolium methyl sulphate	[EMIM][MeSO_4_]
1-ethyl-3-methyl imidazolium ethyl sulfate	[EMIM][EtSO_4_]
1-butyl-3-methylimidazolium hydrogen sulphate	[BMIM][HSO_4_]
1-ethyl-3-methyl imidazolium tetrafluoroborate	[EMIM][BF_4_]
1-ethyl-3-methylimidazolium dicyanamide	[EMIM][DCA]
1-butyl-3-methylimidazolium tetrafluoroborate	[BMIM][BF_4_]
1-butyl-3-methylimidazolium hexafluorophosphate	[BMIM][PF_6_]
1-butyl-3-methylimidazolium dodecanesulfonate	[BMIM][DoS]
1-butyl-3-methylimidazolium bis(trifluoromethanesulfonyl)imide	[BMIM][NTf_2_]
1-hexyl-3-methylimidazolium bis (trifluoromethanesulfonyl) imide	[HMIM][NTf_2_]
1-buty1-butyl-4-methylpyridinum tetrafluoroborate	[BMPy][BF_4_]

**Table 2:** The use of ILs as green solvents, microreactors, and extractants for synthesis and separation purposes.

Application	IL Name	IL Class	Ref.
Separation and extraction of heavy metal ions including Hg^2^^+^ and Cd^2^^+^ from aqueous solution into [C_4_mim][PF_6_]	1-butyl-3 methylimidazolium hexafluorophosphate, [C_4_mim][PF_6_]	RTIL	[33]
Extraction and preconcentration of Cd^2^^+^ and Hg^2^^+^ from aqueous samples into a mixture of different TSILs and a RTIL, [C_4_mim][PF_6_]	Combinations of derivatized imidazolium cations with urea, thiourea, and thioether, mixed with 1-butyl-3 methylimidazolium hexafluorophosphate, [C_4_mim][PF_6_]	TSILs and RTILs	[35]
Synthesis of organic compounds by using ILs as electronic microreactor	1-butyl-3-methylimidazolium tetrafluoroborate, [bmim][BF_4_], and 1-butyl-3-methylimidazolium hexafluorophosphate, [bmim][PF_6_]	RTIL and RTIL	[36]
Dissolution of a softwood lignin in different ionic liquids as aprotic green solvents	1-hexyl-3-methylimidazolium trifluoromethanesulfonate, [hmim][CF_3_SO_3_], 1,3-dimethylimidazolium methylsulfate, [mmim][MeSO_4_], and 1-butyl-3-methylimidazolium methylsulfate [bmim][MeSO_4_]	TSIL, RTIL, and RTIL	[37]
Removal of phenolic compounds such as pentachlorophenol by use of magnetic room-temperature ionic liquid (MRTIL)	trihexyltetradecyl phosphonium etrachloroferrate (III), [3C_6_PC_14_][FeCl_4_]	MRTIL	[38]
Extraction of three alkaloids from lotus leaf	1-hexyl-3-methylimidazolium bromide, ([C(6)MIM]Br)	TSIL	[39]
Extraction and determination of flavonoids from Bauhinia championii	1-butyl-3-methylimidazolium bromide, ([bmim]Br)	TSIL	[40]
Effective extraction of rutin from Chinese medicinal plants	1-butyl-3-methylimidazolium bromide, ([bmim]Br)	TSIL	[41]
Separation of tannins from plant materials	*N*,*N*-dimethylammonium *N*′*N*′-dimethylcarbamate, (DIMCARB)	TSIL	[42]
Extraction and isolation of shikimic acid from Ginkgo biloba leaves	1-butyl-3-methylimidazolium chloride, ([bmim]Cl)	TSIL	[43]
Extraction of Shikonin and *β*,*β’*-dimethylacrylshikonin in Arnebia euchroma (Royle) Johnst	1-hexyl-3-methylimidazolium tetrafluoroborate, [C(6)MIM][BF_4_]	RTIL	[44]

**Table 3:** Summary of the drug delivery reports based on self-propelled ILs’ droplets.

Drug Delivery Application	Chemotactic System (IL Name)	Ref.
Transdermal delivery of Acyclovir (to treat infections caused by certain types of viruses such as cold sores around the mouth)	dimethylimidazolium dimethylphosphate [C_1_mim][(MeO)_2_PO_2_]	[72]
Targeted delivery of Epirubicin (anticancer drug)	trihexyltetradecyl phosphonium chloride ([P_6_,_6_,_6_,_14_][Cl])	[81]
Encapsulating of glucose oxidase alone or in combination with catalase into biocompatible nanoscopic asymmetric polymer vesicles (polymersomes), applications in blood–brain barrier crossing	asymmetric polymersomes: poly [(2-methacryloyl) ethyl phosphorylcholine]–poly[2-(diisopropylamino) ethyl methacrylate] (PMPC-PDPA) and poly[oligo (ethylene glycol) methyl methacrylate] (POEGMA-PDPA)	[82]
Delivery of Piroxicam (a nonsteroidal anti-inflammatory drug), which is sparingly soluble in water	1-hexyl-3-methylimidazolium chloride [Hmim][Cl] and 1-butyl-3-methylimidazolium hexafluorophosphate [Bmim][PF_6_]	[83]
Development of a highly effective oral insulin formulation and delivery	choline and geranate (CAGE) ionic liquid	[84]
Transport live cells protected in alginate capsules as a protective unit along chemical gradients	1-decanol chemotactic droplets in an aqueous medium containing decanoate at high pH by chemical gradient in the external aqueous environment	[85]
Delivery of biologically active anionic pharmaceuticals for anti-inflammatory and anti-coagulant therapy	salicylate decorating poly (2-(trimethylammonium) ethyl methacrylate based on a pharmaceutical ionic liquid	[86,87]
